# Autologous regenerative cell therapy for female stress urinary incontinence: a systematic review and meta-analysis of clinical outcomes

**DOI:** 10.3389/fmed.2026.1861606

**Published:** 2026-06-03

**Authors:** Jiemei Chen, Yaofeng Zhi, Wanmin Liu, Yingchi Fan, Yuehuan Lin, Qian Zhong, Xin Zhang, Xiaohong Ruan

**Affiliations:** 1 Jiangmen Key Laboratory of Precision and Clinical Translation Medicine, Clinical Experimental Center, Jiangmen Engineering Technology Research Center of Clinical Biobank and Translational Research, Jiangmen Central Hospital, Jiangmen, China; 2 Department of Rehabilitation Medicine, The Third Affiliated Hospital, Southern Medical University, Guangzhou, China; 3 Department of Gynecology, Jiangmen Central Hospital, Jiangmen, China; 4 Clinical Transformation and Application Key Lab for Obstetrics and Gynecology, Pediatrics, and Reproductive Medicine of Jiangmen, Jiangmen, China

**Keywords:** cell therapy, female, regenerative cell, stem cell, stress urinary incontinence

## Abstract

**Objective:**

To systematically evaluate the clinical outcomes of autologous regenerative cell therapy for female stress urinary incontinence (SUI).

**Methods:**

A systematic search was conducted in PubMed, Web of Science, EMBASE, Cochrane Library, and CINAHL databases. Data analysis was performed using RevMan and Stata. The primary outcomes were cure and improvement rate. Secondary outcomes included pad test, leakage frequency, questionnaire scores, quality of life, urodynamic parameters, loss to follow-up rate, and adverse events. Subgroup analyses and sensitivity analyses were conducted based on heterogeneity. Publication bias was assessed using funnel plots, begg test, and egger test.

**Results:**

A total of 19 studies encompassing 591 patients were included. The meta-analysis showed that regenerative cell therapy was associated with a pooled cure rate of 41% and a pooled improvement rate of 55% (17 studies). Regenerative cell therapy significantly reduced pad test, decreased leakage frequency, and improved incontinence-specific questionnaires and quality of life. In terms of urodynamic parameters, post-void residual volume was significantly improved, while no significant improvements were observed in maximum urethral closure pressure or maximum flow rate. The loss to follow-up rate and adverse events were no more than 8%. Significant heterogeneity was observed across all outcome measures. Subgroup analysis showed no significant differences and sensitivity analyses confirmed the robustness of the results. Funnel plots suggested potential publication bias, but begg test and egger test were not statistically significant.

**Conclusion:**

Autologous regenerative cell therapy for female SUI achieved a 41% cure rate and 55% improvement rate, improved symptoms and quality of life, and demonstrated favorable short-term safety. However, these findings should be interpreted with caution due to short follow-up, lack of magnetic resonance imaging evidence for regeneration, significant heterogeneity, and low-to-moderate study quality. Future large-sample randomized controlled trials with standardized study protocols are needed to further validate these findings.

**Systematic review registration:**

The registration URL is as follows: https://www.crd.york.ac.uk/PROSPERO, PROSPERO (CRD420261345945).

## Introduction

Stress urinary incontinence (SUI) is defined by the International Continence Society as the involuntary leakage of urine occurring with physical exertion or everyday bodily straining actions, including coughing, sneezing, and exercise. SUI is one of the most common forms of pelvic floor dysfunction in women. Epidemiological studies indicate that approximately 8.2% of adult women worldwide experience some degree of urinary incontinence, among which SUI accounts for approximately 50–70% of cases (1, 2). In European populations, the prevalence of urinary incontinence ranges from 23 to 44%, while in China, the prevalence of SUI among adult women is estimated at 18.9%, with a considerable proportion of affected individuals remain undiagnosed and do not seek medical care (3, 4). Moreover, with the acceleration of population aging, the prevalence of SUI is increasing year by year.

The pathogenesis of SUI is multifactorial. The predominant mechanisms include urethral hypermobility resulting from weakening or relaxation of pelvic floor support structures and intrinsic sphincter deficiency, which may occur independently or in combination (5–7). Common risk factors include advancing age, elevated body mass index, multiple vaginal deliveries, postmenopausal hormonal changes, and chronically increased intra-abdominal pressure. These factors can contribute to damage of pelvic floor muscles and connective tissues, ultimately impairing normal urethral sphincter function (8, 9).

Current management strategies for SUI are generally classified into conservative and surgical approaches. Conservative therapies mainly include pelvic floor muscle training, biofeedback, electrical stimulation, and periurethral injection of urethral bulking agents. However, the efficacy of these methods is often limited, and recurrence rates remain relatively high (7). Surgical treatment, represented by mid-urethral sling procedures, can yield favorable short-term outcomes, yet is associated with invasiveness, a considerable risk of complications, and long-term recurrence (6, 10). Importantly, some patients are not suitable candidates for conventional surgical interventions due to comorbidities, prior surgical failures, or personal preferences. Therefore, investigating less invasive, function-preserving non-surgical or minimally invasive treatment modalities is of substantial clinical significance.

Regenerative medicine has introduced a promising therapeutic paradigm for SUI, such as platelet-rich plasma therapy and stem cells therapy (11, 12). Mesenchymal stem cells (MSCs), owing to their multilineage differentiation potential, immunomodulatory properties, and ability to secrete multiple bioactive factors via paracrine mechanisms, have shown broad application prospects in tissue repair and regeneration (13). Theoretically, periurethral injection of MSCs may facilitate structural and functional reconstruction of the urethral sphincter, while promoting local angiogenesis and nerve regeneration, thereby restoring continence at the pathophysiological level (14). To date, MSCs have been isolated from various tissues, including bone marrow, adipose tissue, skeletal muscle, and umbilical cord blood, and their preliminary efficacy in improving urethral sphincter function and alleviating SUI symptoms has been demonstrated in both animal studies and human trials (15–17).

Although several recent clinical trials have evaluated the safety and efficacy of autologous stem cell injections for female SUI, existing evidence is limited by generally small sample sizes and substantial heterogeneity in cell sources, preparation methods, injection doses, and follow-up durations, which hampers direct comparisons across studies. Prior meta-analyses have attempted to synthesize this field systematically. In 2023, Mariotti et al. pooled data from 12 clinical trials and reported a 41% complete continence recovery rate. However, that analysis included both male and female patients and showed significant heterogeneity (18). In 2021, Huang et al. reported a cure rate as high as 82% in the myocyte-plus-fibroblast subgroup for female patients, yet the included studies involved mixed cell types and some trials had extremely small sample sizes (19). Moreover, these meta-analyses primarily focused on cure and improvement rates, with less attention given to objective incontinence-related outcomes and quality of life (QoL).

Nevertheless, prior meta-analyses have several methodological limitations. First, male SUI most commonly occurs after prostate surgery and differs substantially from female SUI in etiology and pathophysiology. Therefore, pooling male and female populations may introduce confounding bias. Second, previous studies have not comprehensively assessed the effects of regenerative cell therapy on objective urodynamic parameters, questionnaire-based symptom scores, or QoL. Third, there has been insufficient quantitative synthesis of treatment-related adverse events and loss to follow-up rates. In addition, several new clinical studies have been published in recent years, providing an opportunity to update the systematic evaluation (12, 20, 21).

Considering the above, the present meta-analysis exclusively includes female SUI patients and aims to systematically evaluate the clinical outcomes of autologous regenerative cell therapy, including cure rate, improvement rate, pad test, urodynamic parameters, QoL, and adverse events. Compared with previous meta-analyses, the strengths of this study include: (1) strict restriction to female patients to minimize confounding bias attributable to etiological and mechanistic differences; (2) systematic incorporation of the most recent clinical trial evidence up to 2026; and (3) the first meta-analytic quantification of the effects of regenerative cell therapy on urodynamic parameters, incontinence symptom assessments, and QoL. Furthermore, this study integrates loss to follow-up and adverse event incidence to provide a more complete assessment of safety, with the intent to offer clinically relevant evidence-based guidance for future research and practice in this field.

## Methods

### Data sources and searches

This meta-analysis adhered to the guidelines outlined in the Preferred Reporting Items for Systematic Reviews and Meta-Analyses (PRISMA) (22). A systematic literature search was conducted across multiple databases, including PubMed, Web of Science, EMBASE, Cochrane Library, and CINAHL, covering articles published from the inception of each database up to March 15, 2026. The following MeSH terms were employed: ‘Transplantation, Autologous’ [Mesh]; ‘Stem Cells’ [Mesh]; ‘Cells’ [Mesh]; and ‘Urinary Incontinence, Stress’ [Mesh]. These terms were combined using the Boolean operators ‘AND’ or ‘OR’. The details of the search strategy were provided in Supplementary Table 1 (PubMed as an example). After removing duplicate records, the titles and abstracts of the remaining citations were screened to assess their eligibility for inclusion. Full-text articles were then thoroughly reviewed to determine final eligibility based on predefined inclusion criteria. The protocol for this meta-analysis was registered in PROSPERO (CRD420261345945).

### Inclusion criteria

Articles were selected based on the PICOS framework. ① Participants: female SUI patients. ② Intervention: autologous cell therapy, including various regenerated cells and stem cells. ③ Comparator: Placebo, no treatment, other non-cell therapies, or absent (in single-arm studies). ④ Outcome: Clinical outcomes comprised cure rate, improvement rate, pad test, validated incontinence-specific questionnaires, QoL, urodynamic assessment, and incidence of adverse events. ⑤ Study design: Randomized controlled trials (RCTs), prospective studies, and single-arm studies were included.

### Exclusion criteria

Exclusion criteria were as follows: (1) the study subjects were animals. (2) Articles with no available data or duplicate date. (3) Non-English articles.

### Outcome measures

The primary outcomes of this meta-analysis were the cure rate and the improvement rate. Secondary outcomes included: (1) pad test; (2) frequency of urinary leakage episodes; (3) incontinence-specific questionnaires; (4) QoL; (5) urodynamic parameters; (6) loss to follow-up rate; and (7) incidence of adverse events. The urodynamic outcomes assessed were maximum flow rate (Qmax), maximum urethral closure pressure (MUCP), and post-void residual urine volume (PVR). Adverse events included procedure-related adverse reactions (associated with tissue harvesting) and cell therapy-related adverse reactions.

### Data extraction and analysis

Two independent authors (W. M. L and Q. Z) performed data extraction and assessed the risk of bias. Any disagreements were resolved by consulting a third author (X. Z). The extracted data included publication year, first author, participant characteristics, intervention types, and outcome data.

RevMan 5.3 software and Stata 17.0 software were used for data analysis. Heterogeneity was assessed using the Q statistic and the I^2^ statistic. The primary effect size was the pooled proportion with its corresponding 95% confidence interval (CI) for outcome measures. A random-effects model was applied when heterogeneity was significant (I^2^ > 50%), whereas a fixed-effects model was used when heterogeneity was low (I^2^ ≤ 50%). Statistical significance was set at *p* < 0.05. Forest plots were generated to illustrate the pooled results. Publication bias was assessed using funnel plots, along with begg and egger tests, with a *p* value > 0.05 indicating no evidence of publication bias.

Sensitivity analysis was performed to evaluate the robustness of the primary findings. First, a leave-one-out sensitivity analysis was conducted by sequentially excluding each study and re-running the meta-analysis. The pooled cure rate and its 95%CI were recalculated after each exclusion, and the I^2^ statistic was used to assess heterogeneity. In addition, two pre-specified sensitivity analyses were performed: (1) excluding studies with zero events, and (2) excluding small-sample studies (total sample size < 20). These analyses were conducted to assess the potential impact of extreme values and small-study effects on the pooled estimates.

### Quality assessment

The quality of the included single-arm studies was independently assessed by two authors (W. L and Q. Z) using the Joanna Briggs Institute (JBI) Critical Appraisal Checklist for Case Series. The checklist comprises 10 criteria, including: (1) clear definition of inclusion criteria; (2) consecutive recruitment of participants; (3) complete reporting of baseline characteristics; (4) objective and standardized outcome measurement; (5) adequate follow-up; (6) reliability and validity of outcome measures; (7) appropriateness of statistical analysis; (8) reporting of loss to follow-up; (9) documentation of adverse events; and (10) reliability of study conclusions. Each criterion was rated as “yes” (low risk of bias), “no” (high risk of bias), or “unclear” (insufficient information). The overall quality score was calculated as the sum of scores across all criteria. Any disagreements were resolved by discussion or by consulting a third author (X. H. R). The results of the quality assessment are presented in both tabular and graphical formats.

## Result

### Search results and study characteristics

A total of 453 articles were retrieved from the databases. After removing duplicates and screening titles, abstracts, and full texts against the inclusion/exclusion criteria, 21 studies were initially identified. However, two studies (12, 23) were excluded because effective data could not be extracted, resulting in the final inclusion of 19 studies (20, 21, 24–40), encompassing a total of 591 patients.

**Figure 1:**
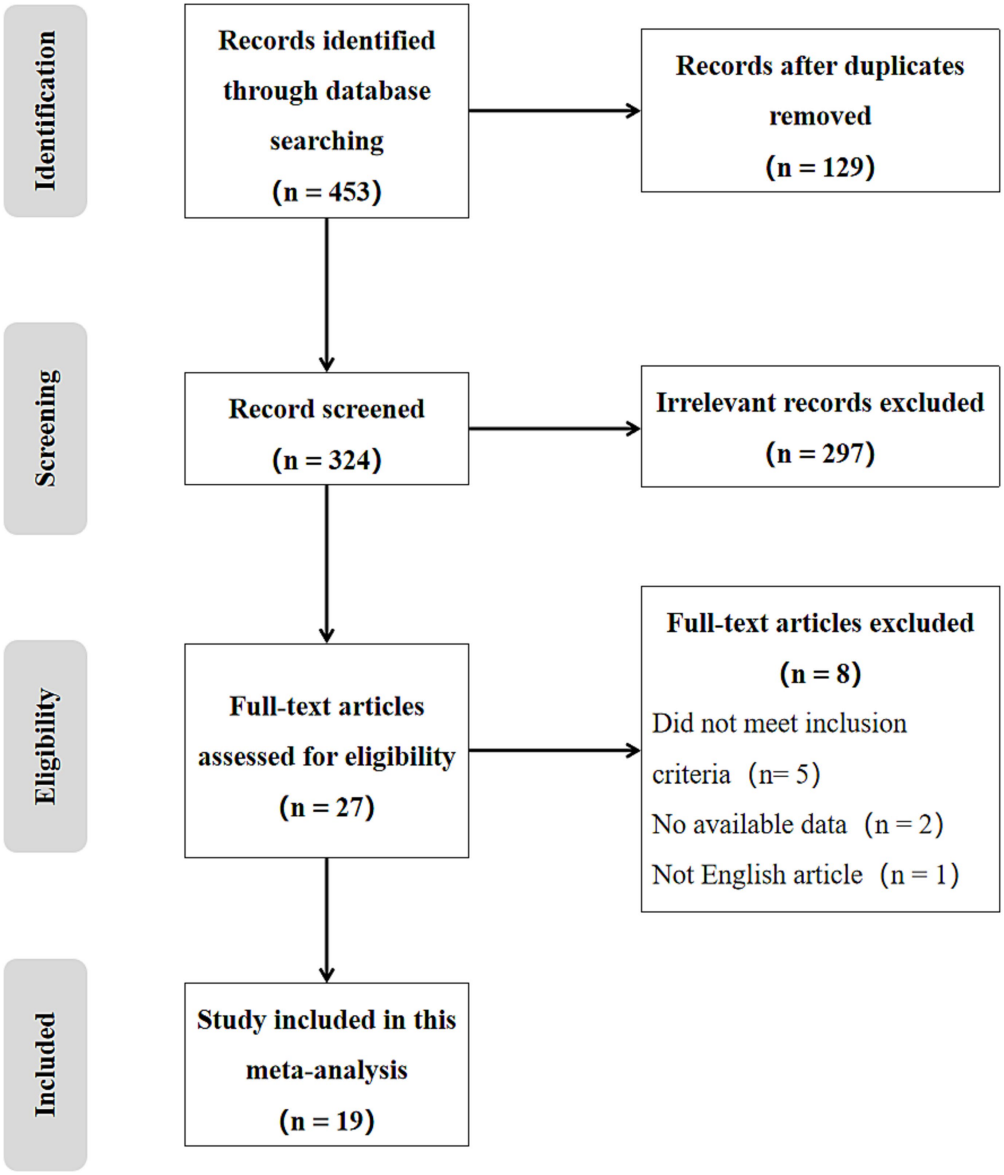
Study flowchart.

Of the 19 included studies, only one (28) was RCTs and the remaining 18 studies were either single-arm studies (without a control group) or comparative studies in which the control group also received cell therapy (e.g., different cell types or different doses). The sample size across studies ranged from 5 to 123 patients. Regarding the source of stem cells, 15 studies utilized muscle-derived stem cells (MDSCs), three studies employed non-muscle-derived stem cells, and one study directly compared the two types. The detailed literature selection process is illustrated in Figure 1, and the basic characteristics of the included studies are summarized in Table 1.

**Table 1:** Overview of the included studies.

Study	Country	Number of participants	Age/yr	BMI kg/m^2^	Disease duration	Cell type	Time of outcome assessment after intervention	Outcome indicator
Blaganje 2013 (24)	Slovenia	38	52	26.6	/	MDSCs	6 months	①②
Carr 2008 (26)	Canada	8	42–65	< 30	> 1 yr	MDSCs	12 months	①②
Carr 2013 (25)	USA	38	50	25.9	> 1 yr	MDSCs	18 months	①②③④⑤
Dutta 2025 (21)	USA	8	53.4	31	/	MDSCs	12 months	③④⑤
Gräs 2014 (27)	Denmark	31	52	24.5	/	MDSCs	12 months	①②④⑤⑦⑧⑨
Jankowski 2018 (28)	USA	93	51.4	26.4	/	MDSCs	12 months	①②
Kuismanen 2014 (29)	Finland	5	59.2	30.2	/	Adipose-derived stem cells	12 months	①②③⑤
Lee 2009 (30)	Korea	39	51.5	/	/	Cord blood stem cells	12 months	①②
Mitterberger 2007 (31)	Austria	123	62.8	/	/	MDSCs	12 months	①②⑤⑥⑦⑨
Mitterberger 2008 (32)	Austria	20	49.8	/	/	MDSCs	12 months	①②⑤⑥⑦⑨
Peters 2014 (33)	USA	80	55	27.8	> 0.5 yr	MDSCs	12 months	①②
Sèbe 2010 (34)	France	12	58	27.6	/	MDSCs	12 months	①②③④⑥
Sharifiaghdas 2016 (35)	Iran	10	29.2	/	/	MDSCs	12 months	①②③⑧
Sharifiaghdas 2019 (36)	Iran	20	51.05	/	/	MDSCs	12 months	①②⑤⑦
Shirvan 2012 (37)	Iran	9	48.88	/	/	Autologous total nucleated cells	6 months	①②⑥
Souza 2026a (20)	Brazil	11	56.6	33.7	/	MDSCs	12 months	①②③⑥
Souza 2026b (20)	Brazil	9	49	28.9	/	marrow-derived mesenchymal stem cells	12 months	①②③⑥
Stangel-Wojcikiewicz 2013 (38)	Poland	16	56.75	46.05	82.5 ± 59.6 months	MDSCs	24 months	①②⑧
Stangel-Wojcikiewicz 2016 (39)	Poland	16	/	/	/	MDSCs	24 months	⑥
Yiou 2013 (40)	France	5	62.5	/	/	MDSCs	3 months	③⑤⑥⑧

MDSCs, muscle-derived stem cells. ① cure rate; ② improvement rate; ③ pad test; ④ frequency of urinary leakage episodes; ⑤ incontinence-specific questionnaires; ⑥ quality of life; ⑦ maximum flow rate; ⑧ maximum urethral closure pressure; ⑨ post-void residual urine volume; MDSCs, muscle-derived stem cells.

### Meta-analysis results

#### Primary outcomes

**Figure 2:**
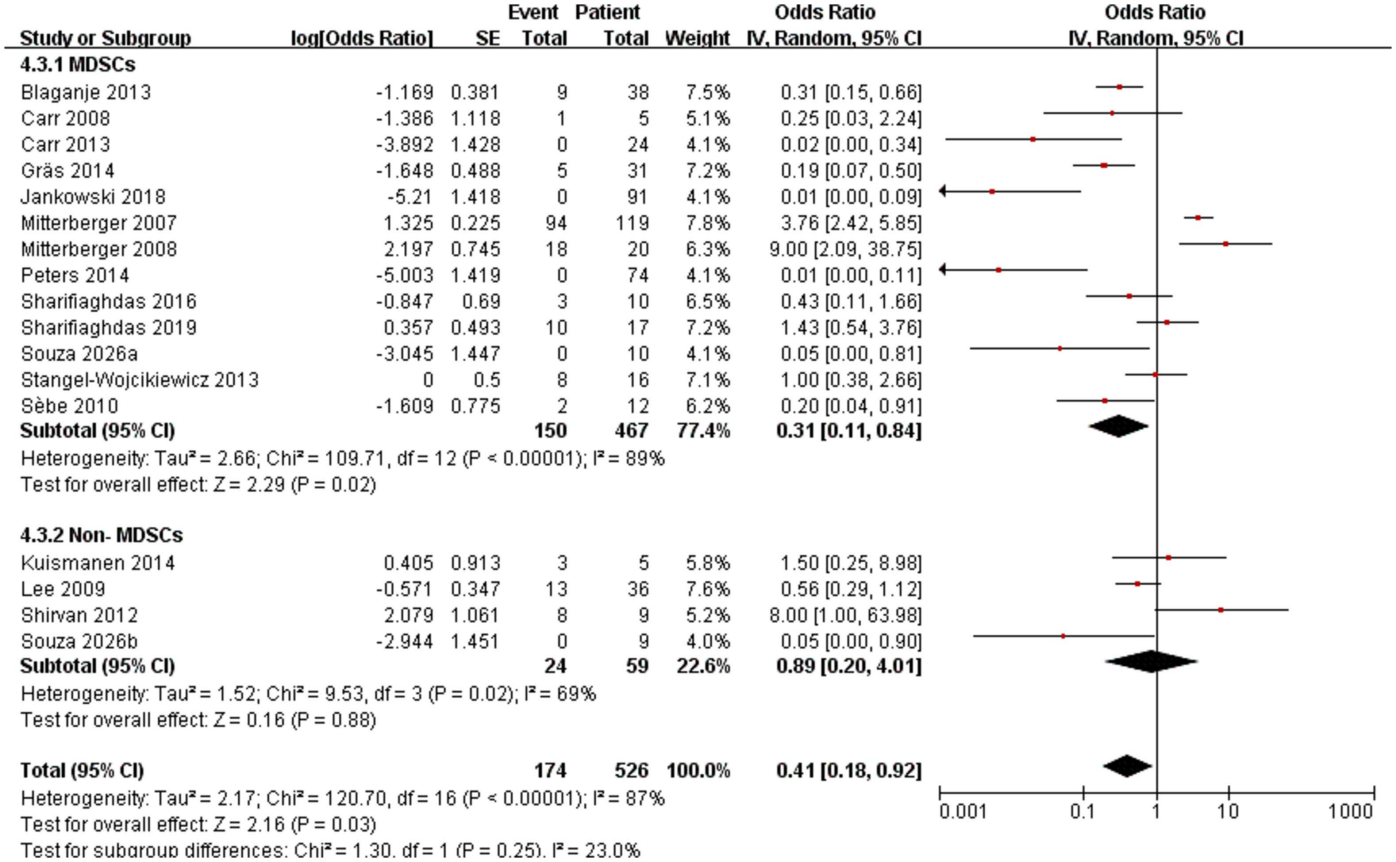
Forest plot of cure rate.

**Figure 3:**
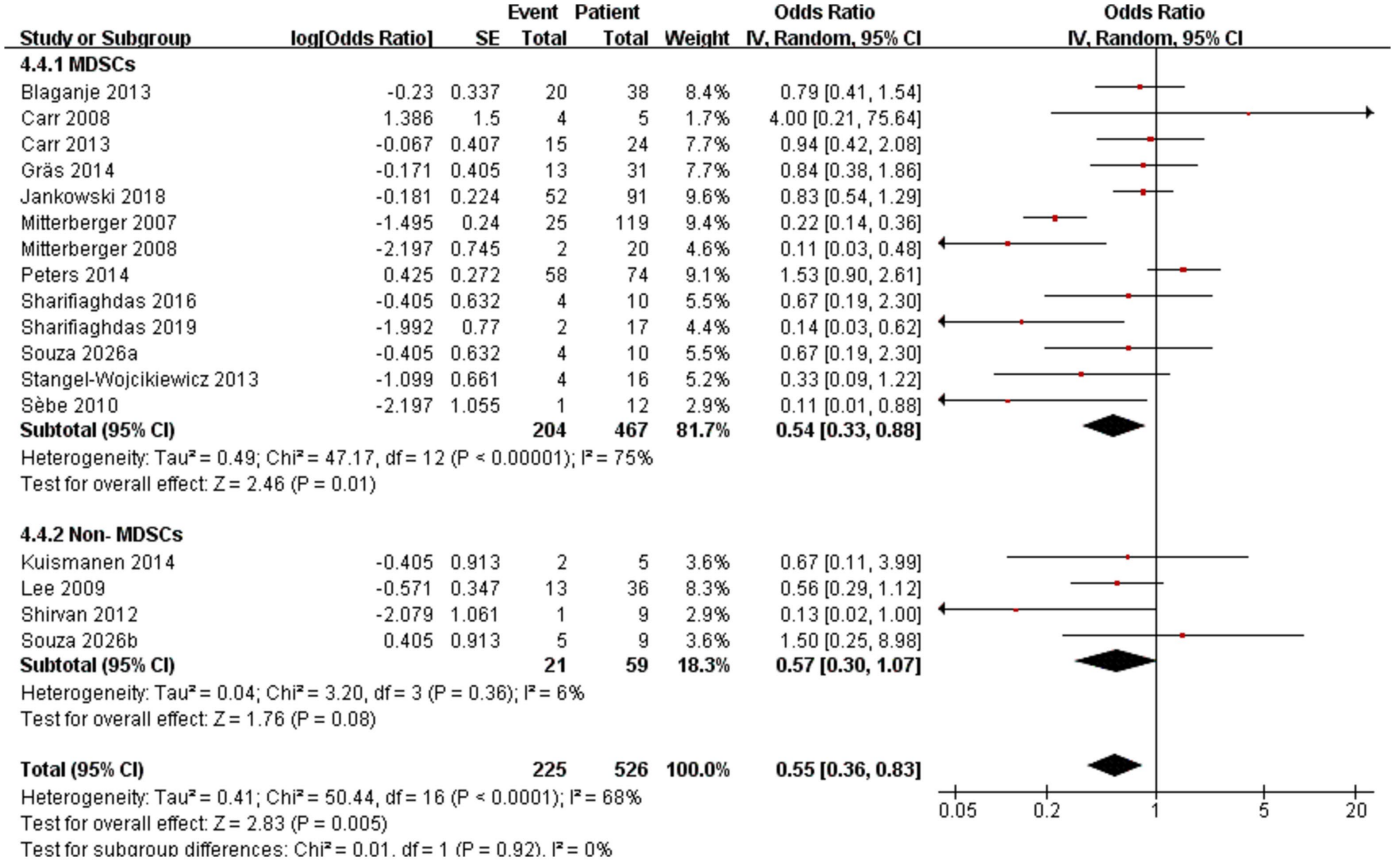
Forest plot of improvement rate.

Seventeen studies reported the cure rate and improvement rate. A summary of the definitions of cure and improvement employed by the individual studies is available in Supplementary Table 2. The meta-analysis showed that regenerative cell therapy was associated with a pooled cure rate of 41% (OR = 0.41, 95% CI: 0.18 to 0.92, *p* = 0.03; Figure 2) and a pooled improvement rate of 55% (OR = 0.55, 95% CI: 0.36 to 0.83, *p* = 0.005; Figure 3) in female SUI patients. Significant heterogeneity was detected for both outcomes (cure rate: I^2^ = 87%, *p* < 0.00001; improvement rate: I^2^ = 68%, *p* < 0.0001).

#### Secondary outcomes

**Figure 4:**
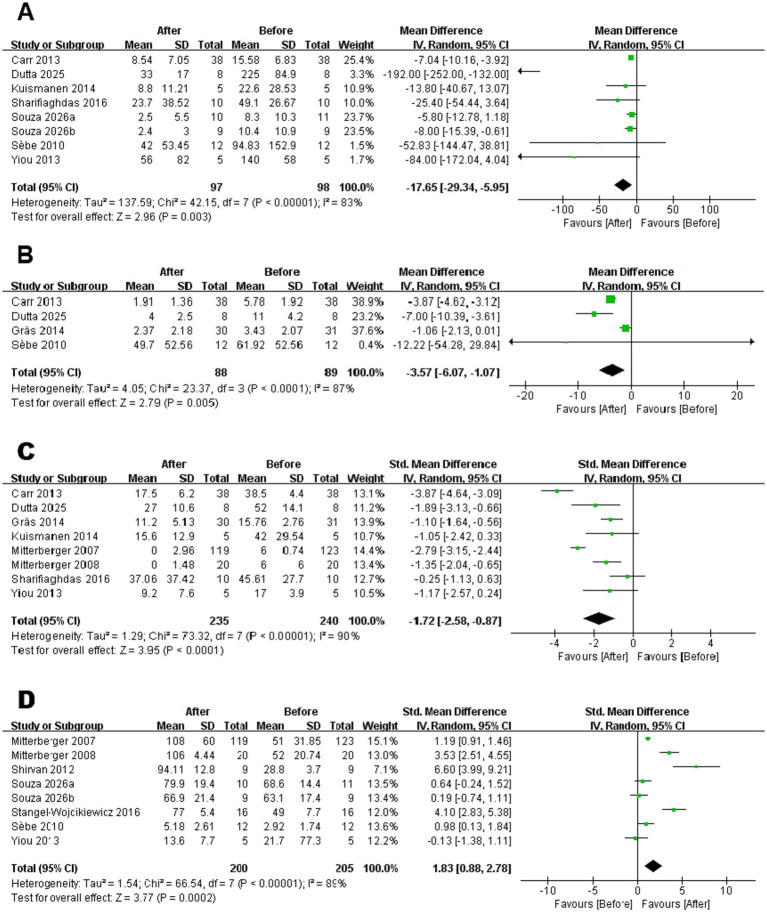
Forest plot of **(A)** pad test, **(B)** leakage frequency, **(C)** incontinence-specific questionnaire, **(D)** QoL.

The meta-analysis showed that regenerative cell therapy significantly reduced pad test (8 studies, MD = −17.65, 95% CI: −29.34 to −5.95, *p* = 0.003; I^2^ = 83%), leakage frequency (4 studies, MD = −3.57, 95% CI: −6.07 to −1.07, *p* = 0.005; I^2^ = 87%), incontinence-specific questionnaire scores (8 studies SMD = −1.72, 95% CI: −2.58 to −0.87, *p* < 0.0001; I^2^ = 90%), and improved QoL scores (8 studies, SMD = 1.83, 95% CI: 0.88 to 2.78, *p* = 0.0002; I^2^ = 89%) compared to baseline (Figure 4).

**Figure 5:**
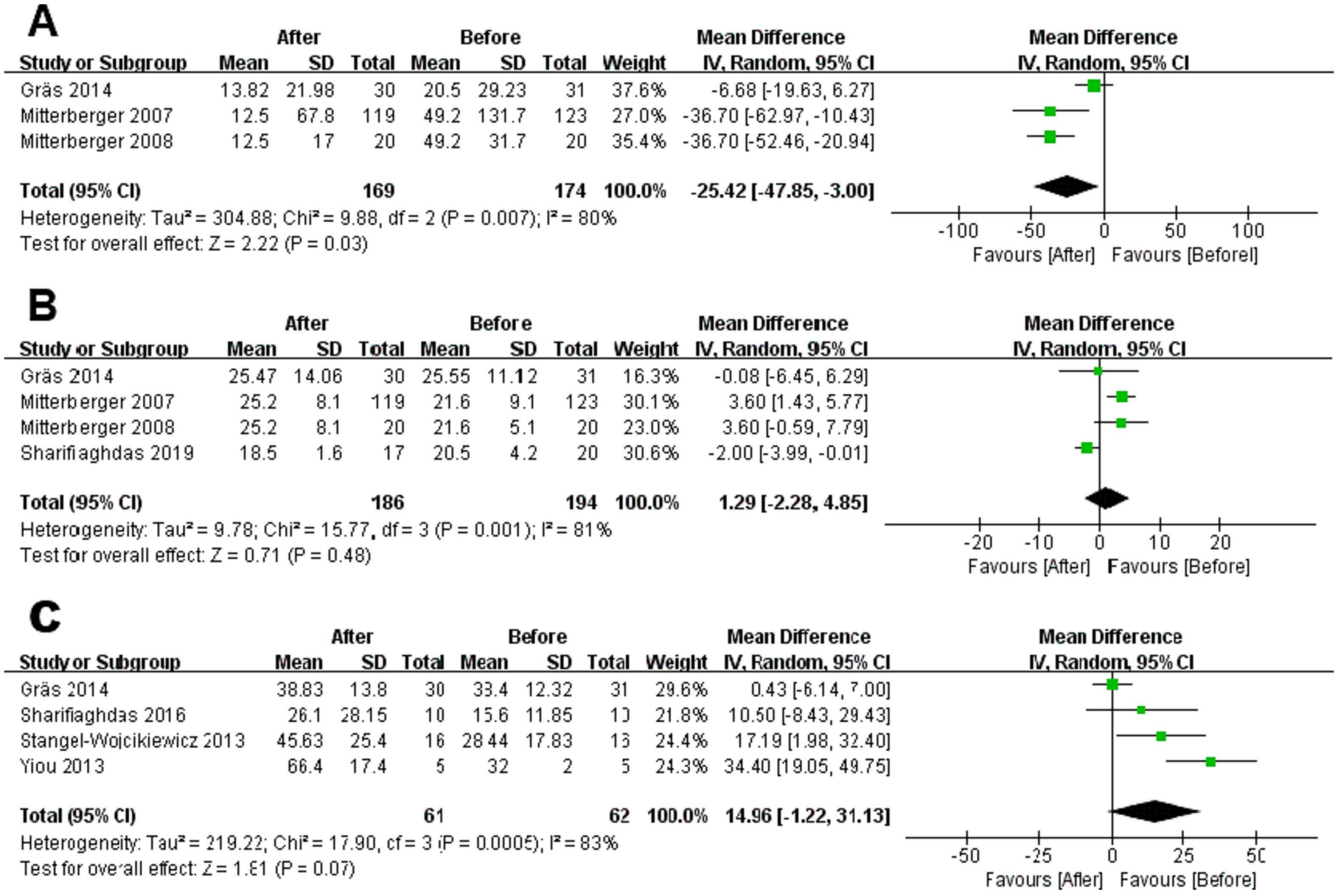
Forest plot of **(A)** PVR, **(B)** Qmax, **(C)** MUCP.

Regarding urodynamic assessment, the meta-analysis demonstrated that regenerative cell therapy significantly improved PVR (3 studies, MD = −25.42, 95% CI: −47.85 to −3.00, *p* = 0.03; I^2^ = 80%). In contrast, no significant improvement was observed for Qmax (4 studies, MD = 1.29, 95% CI: −2.28 to 4.85, *p* = 0.48; I^2^ = 81%) or MUCP (4 studies, MD = 14.96, 95% CI: −1.22 to 31.13, *p* = 0.07; I^2^ = 83%; Figure 5).

### Loss to follow-up rate and adverse events

The meta-analysis showed that regenerative cell therapy was associated with a pooled loss to follow-up rate of 4% (95% CI: 2 to 8%, *p* < 0.0001, I^2^ = 53%), a pooled procedure-related adverse reaction rate of 8% (95% CI: 4 to 16%, *p* < 0.0001; I^2^ = 79%), and a pooled cell therapy-related adverse reaction rate of 7% (95% CI: 4 to 14%, *p* < 0.0001; I^2^ = 68%) in female SUI patients. Procedure-related adverse reactions included pain, bleeding, and infection at the tissue harvest site. Cell therapy-related adverse reactions primarily comprised urinary tract infection and dysuria. All adverse events resolved spontaneously without the need for specific medical intervention.

### Sensitivity analyses and reporting bias

Sensitivity analyses were performed for the primary outcomes (cure rate and improvement rate) to assess the robustness of the findings. Leave-one-out analyses yielded pooled cure rates ranging from 36 to 42% and pooled improvement rates ranging from 38 to 43%, which were consistent with the primary estimates. All results remained statistically significant (*p* < 0.05). Excluding zero-event studies did not change the pooled estimates, while excluding small-sample studies (n < 20) yielded slightly altered rates with wider confidence intervals. These findings suggest that the results were robust. However, I^2^ values remained high across all sensitivity analyses (cure rate >93%; improvement rate >83%), indicating substantial heterogeneity. Detailed results are presented in the Supplementary Table 3.

**Figure 6:**
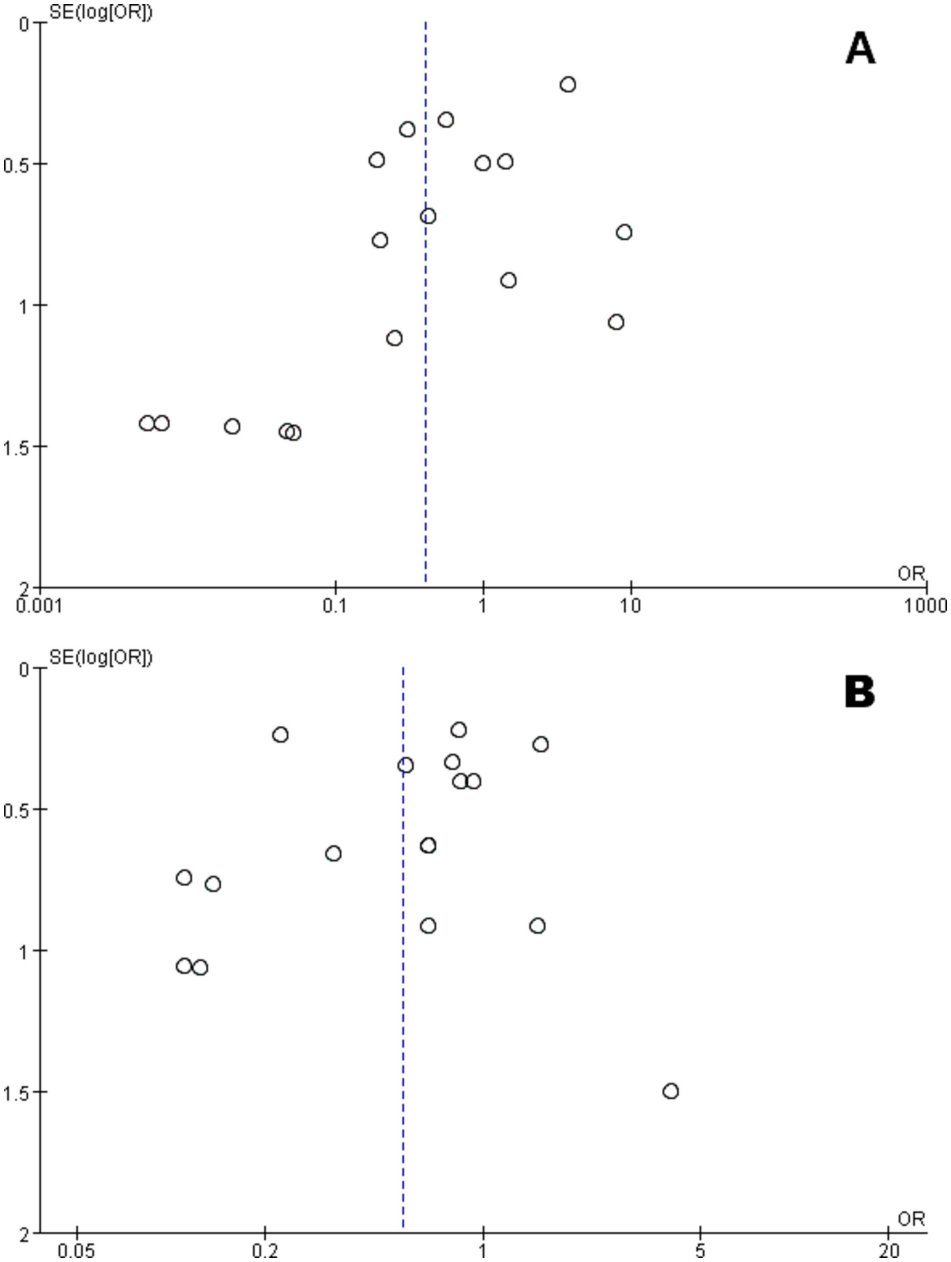
Funnel plot of **(A)** cure rate and **(B)** improvement rate.

Funnel plots indicated publication bias in cure rate and improvement rate (Figure 6), but egger test (*p* values of 0.337 and 0.845, respectively) and begg test (*p* values of 0.621 and 0.364, respectively) suggested no publication bias.

### Subgroup analysis

Subgroup analyses were performed according to cell type to investigate potential sources of heterogeneity for both cure and improvement rates. The results revealed no statistically significant difference between the muscle-derived stem cells (MDSCs) and non-MDSCs subgroups for cure rate (χ^2^ = 1.30, df = 1, *p* = 0.25, I^2^ = 23.0%; Figure 2). Likewise, for improvement rate, no significant difference was detected between the two subgroups (χ^2^ = 0.01, df = 1, *p* = 0.92, I^2^ = 0%; Figure 3).

### Quality assessment

The quality of the included studies was assessed using the JBI Critical Appraisal Checklist for Case Series. The overall methodological quality of the included studies was considered moderate. Most studies clearly defined their inclusion criteria and used objective outcome measures (e.g., pad test, urodynamic parameters, and validated questionnaires). However, consecutive recruitment was not explicitly reported in several studies, and loss to follow-up was not consistently documented.

## Discussion

Given the high adherence demands of conservative treatment and the risks and failure rates associated with surgery, regenerative therapy remains clinically valuable. This study systematically assessed the clinical efficacy and safety of autologous regenerative cell therapy for female SUI. Overall, regenerative cell therapy was associated with a 41% complete continence restoration rate and a 55% improvement rate. It also reduced pad test, leakage frequency, and incontinence-specific questionnaire scores, while improving QoL. In urodynamic outcomes, PVR decreased significantly. However, improvements in MUCP and Qmax were not statistically significant. Regenerative cell therapy showed a low loss to follow-up rate and a low incidence of treatment-related adverse events, most of which were mild and self-limiting.

The 41% cure rate observed here aligns with prior evidence. Mariotti et al. (18) pooled 12 clinical trials and reported a 41% complete continence restoration rate, suggesting that complete continence may be relatively consistent across studies from different periods. Nevertheless, their analysis included both male and female patients and did not stratify by sex, limiting sex-specific inference. The present meta-analysis restricted inclusion to female SUI, thereby reducing confounding related to differences in the underlying mechanisms of male versus female SUI (41). Given the fundamental differences in etiology and anatomy, the restriction to female patients increases clinical specificity.

In contrast, Huang et al. (19) reported more favorable efficacy in the myocyte-plus-fibroblast subgroup, with cure rates of 82–89% and improvement rates of 92–97% for female patients, which were higher than the pooled estimates in this study and those reported by Mariotti et al. (18). Variability in patient selection criteria, cell sourcing and preparation, injection protocols, and outcome definitions may contribute to these discrepancies. Additionally, some studies may have used more permissive definitions of “cure,” and concurrent postoperative pelvic floor rehabilitation could potentially influence outcome assessment.

Our findings indicate that the cure and improvement rates of regenerative cell therapy were lower than those of surgery but comparable to conservative treatment. The objective cure rate of mid-urethral sling surgery can reach 80 to 94%, while the complete continence rate of artificial urinary sphincter can reach 72% (42, 43). Regarding conservative treatment, pelvic floor muscle training achieves cure rates of 20–50%, whereas electrical stimulation and biofeedback electrical stimulation show better improvement in incontinence scores (44). The cure and improvement rates of regenerative cell therapy in this study fall within the range of conservative treatments and are slightly better than pelvic floor muscle training alone. However, direct comparison should be made with caution, as cell therapy recipients often represent more complex cases, such as those who failed conservative therapy or were unsuitable for surgery, and some trials included patients with multiple prior interventions (45).

This meta-analysis also provides valuable insights by systematically quantifying effects of regenerative cell therapy across multiple core outcome domains. Objectively, the pad test is widely used to assess SUI severity. Regenerative cell therapy significantly reduced pad test weight and leakage frequency, indicating decreased symptom burden and reduced pad dependence. These objective improvements were supported by patient-reported outcomes. Pooled results further showed significant improvements in disease-specific questionnaire scores and overall QoL (46). The significant improvement in QoL suggests that even when patients do not achieve complete continence, the relief of symptoms is sufficient to translate into meaningful daily benefits (46, 47).

Mechanistically, regenerative cell therapy is intended to promote regeneration rather than provide purely supportive effects (48). However, as highlighted in a critical analysis of this evidence (49), the current studies lack robust objective outcome measures, especially magnetic resonance imaging. In this analysis, while PVR decreased significantly, MUCP and Qmax did not show meaningful improvement. If genuine sphincter regeneration had occurred, an increase in MUCP would be expected. The lack of significant benefit on this parameter may indicate that observed clinical gains could partially reflect a “bulking effect” rather than robust sphincter regeneration. Souza et al. (20) also reported subjective improvement in some participants without significant changes in objective urodynamic parameters and terminated the trial early due to limited efficacy. The clinical meaning of reduced PVR should therefore be interpreted as hypothesis-generating and warrants further confirmation. Future studies could incorporate more sensitive urodynamic assessments, such as abdominal leak point pressure, together with imaging modalities, to evaluate structural changes in the sphincter.

Clinical outcomes may also be influenced by the biological activity of MSCs. The effects of stem cells are thought to occur predominantly via immunomodulation and trophic activity, processes typically triggered by acute injury signals (e.g., trauma, ischemia, or inflammation) that stimulate chemokine and cytokine release (50). The injected periurethral environment is often characterized by chronic scarring and relative quiescence, which may reduce MSCs activation and engraftment in SUI patients. Moreover, needle-related tissue injury during injection may be insufficient to induce a robust cellular response.

Safety findings in this meta-analysis were summarized quantitatively, including loss to follow-up and adverse event incidence. Loss to follow-up was 4%, suggesting good retention in clinical trials. Procedure-related adverse events occurred in 8%, and cell therapy-related adverse events were reported in 7% (primarily urinary tract infection and dysuria). All reported adverse events were mild and self-limiting, and no tumor formation was no tumor formation was reported during the longest follow-up periods. However, the conclusions regarding safety should be limited to short-term outcomes, as most included studies reported follow-up durations of ≤24 months, with some limited to 6–12 months. The long-term *in vivo* persistence of mesenchymal stem cells, potential risks of malignant transformation, and the possibility of ectopic differentiation require further clarification through longer-term follow-up studies (51). In addition, differences in cell product preparation and quality control procedures across trials may limit the generalizability of safety conclusions.

Moderate-to-significant heterogeneity was observed across primary and secondary outcomes. Sensitivity analyses suggested that the pooled effect estimates were generally robust. However, heterogeneity did not decrease substantially, indicating that it was unlikely driven by any single study. Subgroup analyses by cell type (MDSCs vs. non-MDSCs) showed no statistically significant differences in cure or improvement rates, suggesting that cell source may not be a major determinant of heterogeneity. Potential sources of heterogeneity may include differences across studies in outcome definitions (e.g., variable criteria for “cure” and “improvement”), cell preparation and treatment protocols (e.g., cell dose, injection technique), and baseline patient characteristics (e.g., age, disease severity, prior treatments). With regard to publication bias, funnel plot asymmetry suggested potential bias, but both begg and egger tests were not statistically significant, providing no definitive evidence of publication bias.

### Limitation

This study has several limitations. First, the overall methodological quality of the included studies was moderate, which substantially limits the strength of the conclusions. Most included studies were single-arm or non-randomized designs, with only one RCT identified. This should be emphasized more clearly, as the lack of control groups and randomization increases the risk of bias and reduces the level of evidence. Second, both clinical and statistical heterogeneity were observed across studies. Although subgroup and sensitivity analyses were performed, some sources of heterogeneity could not be fully explained. Third, most studies had relatively small sample sizes, which may increase the risk of small-study effects and reduce the precision of pooled estimates. Fourth, it is also important to acknowledge that the definitions of “cure” and “improvement” varied across the included studies. This inconsistency may affect the validity of the pooled estimates and should be clarified. Fifth, follow-up durations in most studies were relatively short, thereby precluding reliable conclusions regarding the long-term safety and durability of regenerative cell therapy. Safety findings should therefore be interpreted as reflecting only short-term outcomes.

To address these limitations, future studies should prioritize several key improvements. Large-sample, multicenter, well-designed RCTs with blinded assessment and standardized outcome definitions are urgently needed. Standardized cell processing protocols and comprehensive reporting of cell source, dose, and injection procedures should be established to reduce inter-study heterogeneity. More objective and multidimensional efficacy evaluation, incorporating urodynamic parameters, standardized pad tests, and patient-reported outcomes, should be adopted. Finally, longer-term follow-up is necessary to assess the durability and long-term safety of regenerative cell therapy.

## Conclusion

This meta-analysis systematically evaluated the clinical outcomes of autologous regenerative cell therapy for female stress urinary incontinence. The results showed that regenerative cell therapy achieved complete continence restoration in 41% of patients and symptom improvement in 55% of patients, while significantly reducing pad test weight, decreasing leakage frequency, and improving disease-specific symptoms and QoL. Regarding safety, regenerative cell therapy demonstrated a favorable short-term safety profile, with a low loss to follow-up rate and a low and manageable incidence of adverse events. Furthermore, this study found that autologous regenerative cell therapy improved post-void residual volume but had no significant effect on MUCP or Qmax.

Notably, significant heterogeneity existed across the available evidence, and the conclusions of this meta-analysis should be interpreted with caution due to the methodological limitations of the original studies. Future research should focus on conducting well-designed, large-sample randomized controlled trials, establishing standardized cell preparation and evaluation systems, and implementing long-term follow-up to confirm long-term safety and durability of efficacy.

## Data Availability

The raw data supporting the conclusions of this article will be made available by the authors, without undue reservation.
